# Facial Contouring by Using Dermal Fillers and Botulinum Toxin A: A Practical Approach

**DOI:** 10.1007/s00266-019-01361-1

**Published:** 2019-04-05

**Authors:** Lisandro Farolch-Prats, Celeste Nome-Chamorro

**Affiliations:** 1 Nu Clinic, Carretera de Cerdanyola, 79-81, 2do 3era, 08172 Sant Cugat del Vallès, Barcelona Spain; 2 Centro Médico Kineergomed, Linares, Chile

**Keywords:** Plastic aesthetic, Dermal filling implants, Botulinum toxin A, Facial contouring, Morphology, Balance

## Abstract

**Background:**

The perception of an attractive face is largely subjective. The purpose of this paper is to provide an insight and a practical approach to facial contouring management with hyaluronic acid (HA) implants and botulinum toxin A.

**Methods:**

This study is presenting the clinical experience of the authors regarding facial contouring. After a careful medical history, patients underwent an exhaustive aesthetic assessment that includes photographs and videos. Realistic treatment goals were discussed and agreed with the patient. Comprehensive treatment strategies for facial contouring, including HA implants and/or botulinum toxin A injections, were selected according to the patient needs.

**Results:**

Based on the MD codes^®^, developed by Mauricio de Maio, these treatment strategies have been adapted to six different basic categories of facial shapes, namely round, square, triangular, inverted triangle, rectangle, oval and oblong faces. The incidence of complications was low and, in all the cases, was mild (edema, erythema and local ecchymosis), of limited duration, and was resolved without sequela.

**Conclusions:**

The current article presented the personal experiences of the authors on a specific subject, and this fact should be considered when interpreting data from this paper. As other aesthetic treatments, facial contouring should be focused on the patient needs and to select a specific aesthetic approach according to different facial shapes. Finally, it is essential to have a good understanding of the potential associated complications, because it will help the specialist to take the necessary precautions to prevent them, and if they ever arise, to be able to deal with them effectively.

**Level of Evidence IV:**

This journal requires that authors assign a level of evidence to each article. For a full description of these Evidence-Based Medicine ratings, please refer to the Table of Contents or the online Instructions to Authors www.springer.com/00266.

## Introduction

The perception of an attractive face is largely subjective, with ethnicity, age, gender, culture and personality influencing average facial traits [1, 2]. The face must be assessed by thorough and judicious analysis conducted by means of a first interview, exhaustive clinical examination as well as supplementary diagnostic examination including photographs, video for dynamic assessments, etc. [3, 4].

There are three fundamental aspects that have to be evaluated during the patient facial assessment and diagnosis: morphology, balance and symmetry.

Although the beauty of the person’s face is determined by the harmony of proportions and symmetry [5], the definition of an attractive and beautiful face is subjective and different factors, such as social, cultural, ethnic and age, should be considered [6].

Facial symmetry might have a positive influence on facial attractiveness for both males and females [7]. Nevertheless, slight asymmetry can give a more natural perception and may personalize the face and not be considered unattractive [8].

Less invasive aesthetic procedures for facial aesthetic beautification [9] and enhancement are evolving continuously.

Over the past two decades, we have seen unprecedented growth in the popularity of elective aesthetic procedures. Furthermore, there has been an increasing demand for less invasive aesthetic procedures, such as dermal filling implants and botulinum toxin A (BoNTA) [10]. As reported by the American Society of Plastic Surgeons in 2012, the aesthetic use of BoNTA and dermal filling implants among specialists increased from 2000 to 2012 by 680% and 205%, respectively [11]. Moreover, the global statistics of the International Society of Aesthetic Plastic Surgery reported an increase, from 2015 to 2016, in the aesthetic use of BoNTA and dermal filling implants of 7% and 18%, respectively [12].

Although less invasive nonsurgical aesthetic procedures, including dermal filling implants and BoNTA injections, are effective and have favorable safety profiles, early and late complications with varying levels of severity may occur [13, 14].

Prevention and treatment of dermal filling implant complications have been widely evaluated in two different expert consensus recommendations [15, 16].

To prevent dermal filling implant complications, Urdiales et al. [15] recommended obtaining an accurate medical history. Additionally, for preventing the incidence of complications, they also recommended using supplementary diagnostic examinations, to evaluate the patient’s health status; a careful preparation of the skin (for preventing of tissue infections), to select the appropriate technique and the right dermal filler according to the patient characteristics, etc.

The purpose of this paper is to provide an insight and a practical approach to facial contouring management with dermal filling implants and botulinum toxin A.

## Methods

This study is presenting the clinical experience of the authors regarding facial contouring.

Written informed consent was signed for all the patients. Patients received full information about the treatment; technique of administration; outcomes; pre- and post-procedure cares; side effects, potential complications and any other additional information required to achieve optimum results. The ethical principles outlined in the Declaration of Helsinki and Good Clinical Practice were followed.

### Medical Assessment of the Patient

The first step is to perform a careful medical history of the patient, paying special attention to history of any medical illnesses and medical interaction that might increase the risk of complications, such as bleeding disorders, uncontrolled hypertension and patients taking anticoagulants.

Exhaustive clinical examination commencing with determining the main complaint and evaluating the medical history remains the foremost essential diagnostic. Aesthetic evaluation of the face must first consider the goals of the patient. Nevertheless, it is essential to stablish realistic goals and agreed upon with the patient.

### Aesthetic Assessment

#### Facial Morphology

Knowledge of basic face structure and face shapes leads to an understanding of the concept of balance.

Facial type assessment is, in many aspects, crucial for the planning and prognosis of esthetic treatment. Different models of facial shape have been proposed over the years.

For evaluating the different facial morphologies, it is important to take into account: forehead width; cheekbone width; and jawline and facial length.

Thus, Rudolf Pöch proposed ten different facial phenotypes that included elliptic, oval, inverted oval, round, rectangle, square, rhomboid, trapezoidal, inverted trapezoidal and pentagonal.

**Fig. 1:**
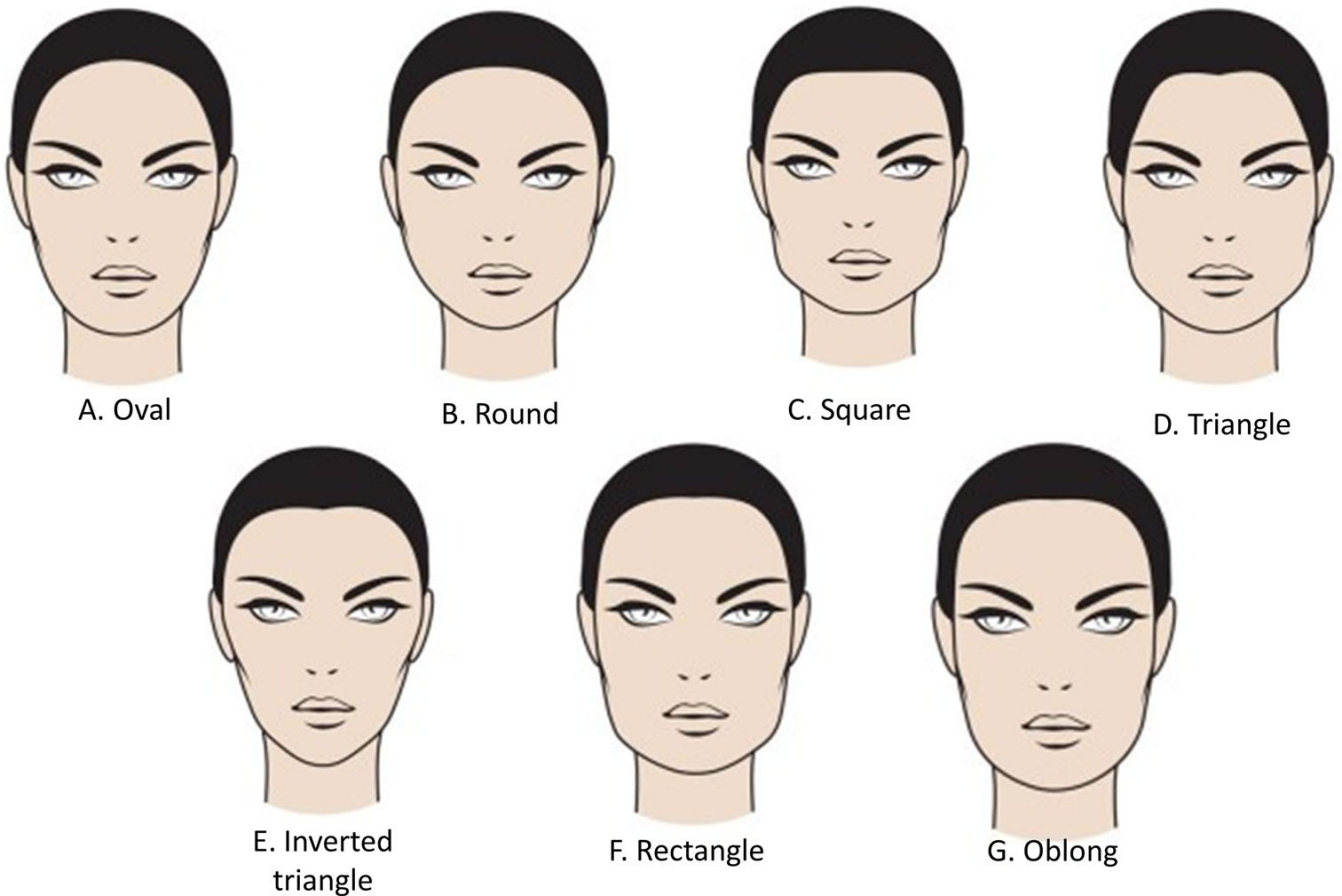
Different types of facial shapes. Attending to its morphology, the face can be divided into different categories, namely oval, round, square, triangle, inverted triangle, rectangle and oblong

Based on the Pöch model [17], it is possible to establish an updated classification of seven different basic categories of facial shapes (Fig. 1):

- Oval (Fig. 1a): This is considered the ideal facial shape because of its balance and overall look of symmetry. It is characterized by a wider appearance in the middle third, which is narrowing slightly toward the chin.
- Round (Fig. 1b): It is a face of smooth lines, without angles, wide and short in length. This face is widest at the cheekbone area and is usually not much longer than it is wide, having a softly rounded jawline, short chin and a rounded hairline over a rather full forehead. Visually, the eyes, mouth and nose are very close together.
- Square (Fig. 1c): This is a wide face with an angular jawline and a square forehead. The lines of this face are straight and angular.
- Triangle (Fig. 1d): It is a face that presents excessive volume in the lower zone, wide jaw and very straight and, sometimes, at the same level of the chin. It is a face that is narrowing at the level of the temples and the forehead.
- Inverted triangle (Fig. 1e): This face has a retracted and narrow jaw, with a prominent chin, wide forehead and broad cheekbones. All these features conferred the characteristic form of inverted triangle
- Rectangle (Fig. 1f): This face shape has straight lines with an angular jawline, similar to that of the square face, but this face is longer
- Oblong (Fig. 1g): This face shape is long and narrow and is very suggestive due to its exoticism. It is an oval of forehead and narrow jaws, long chin, and high and prominent cheekbones.

#### Balance

**Fig. 2:**
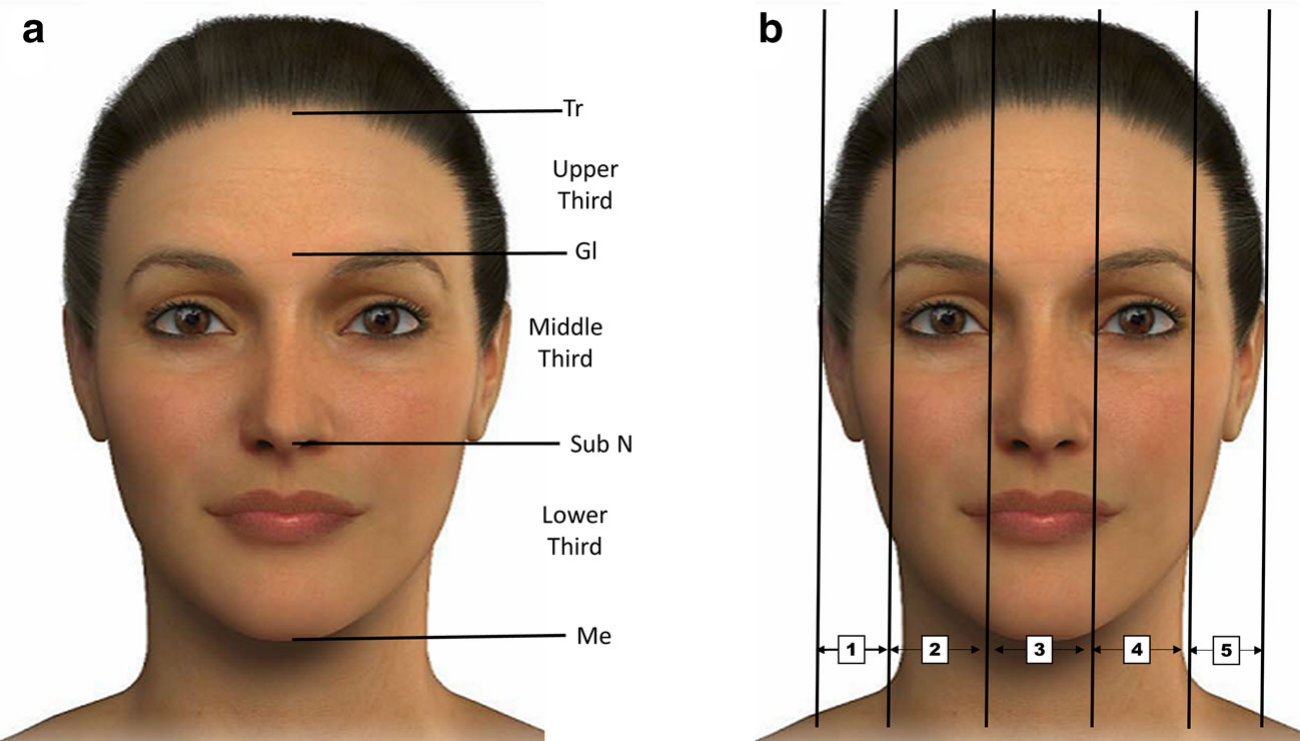
Different divisions of the face. **a** Division of the face into vertical fifths. **b** Division of the face into horizontal thirds. 1 upper third: Tr-Gl; 2 middle third: Gl-subN; 3: lower third: subN-Me. *Tr* Trichion, *Gl* glabella, *Me* menton, *SubN* subnasale. The images of this figure are an Allergan property, and consent was provided for their use in this publication

Different methods have been used to evaluate facial characteristics including anthropometry, photogrammetry, computer imaging and cephalometry [18]. The face may be divided into horizontal thirds (Fig. 2a) and vertical fifths (Fig. 2b) [19]. The facial height is divided into three equal parts: The first third goes from the trichion (Tr) to the glabella (G), the middle third from the G to the subnasal point (subN) and from this point to the menton corresponds to the lower third of the face (Fig. 2a) [19]. On the other hand, the facial width is divided into five equal parts, each part approximately equal to the width of an eye (Fig. 2b) [19].

In the contour observation, the assessment is based on the Ricketts “Esthetic Plane” or “E” plane. Essentially, the “E” plane is a line drawn from the tip of the nose to the tip of the chin [20]. The key issue is to look at how the upper and lower lip relate to that line [20]. Ideally, the lower lip would be 2 mm behind the line, and the upper lip 4 mm behind the line, with variations being normal for patients of different ethnic backgrounds, but with some commonalities applying to all patients [20].

Skeletal malocclusion is a common birth defect that occurs due to the distortion of the maxillary and/or mandibular development that will have a huge impact on the positioning, alignment and health of the primary and permanent teeth [21].

### Retrognathia

Retrognathia is a condition characterized by an abnormal posterior positioning of the mandible relative to the facial structure, which makes it look like the subject has a severe overbite [21].

Retrognathia is associated with: (1) everted lower lip and labial incompetence; (2) marked labiomental furrow due to the depressor anguli oris muscle contraction to help close the mouth; (3) contraction of the mentalis muscle to the labial closure; (4) short neck length; (5) convex facial profile; (6) obtuse angle of mandible; and (7) class II malocclusion (a frequently and challenging encountered problem in orthodontic patients).

### Prognathism

Prognathism is a common skeletal facial abnormality, characterized by a bulging out (protrusion) of the jaw [22].

Prognathism is associated with: (1) concave facial profile; (2) protuberance of the lower third and skeletal deficiency of the middle third; (3) class III malocclusion (one of the most severe maxillofacial deformities); (4) paranasal deficiency with narrow alar base; and (5) upper lip retracted and with thin vermilion.

#### Symmetry

A certain degree of facial asymmetry is a common in the general population. As previously mentioned in Introduction, slight asymmetry provides a more natural perception of the face and, under no circumstances, should such a level of asymmetry be considered unattractive.

It has been reported that nonpathologic facial asymmetry is likely to exhibit laterality [23, 24]. It was suggested that the right hemiface is usually wider than the left one [25].

#### Technique: Facial Contouring with Hyaluronic Acid Implants and Botulinum Toxin A

Selection of HA implant is based on the indication and site of placement.

Our treatment strategy is based on the MD codes^®^, which were developed by Mauricio de Maio [26–28].

The technique utilizes the Juvéderm Vycross^®^ (VYC) (Allergan, Irvine, CA, USA) collection of non-permanent HA dermal fillers [29]. These hyaluronic acid dermal fillers work in specific ways to volumize and give structure to the face:

- For lifting and restoring volume, promoting an improvement in the structural foundations and facial contour, a high-density HA implant (VYC-20L) is injected deeply into supraperiosteum.
- To treat medium-deep depression, we used a mid-density hyaluronic acid filler injected subdermally.
- For treating the periorbital area, as well as the area around the lip, giving finished facial refinements, a low-density hyaluronic acid filler with a water-like consistency filler is selected.

As regards the Botulinumtoxin A, the technique utilizes Vistabel/Botox Cosmetics^®^ (OnabotA) (Allergan, Irvine, CA, USA).

**Fig. 3:**
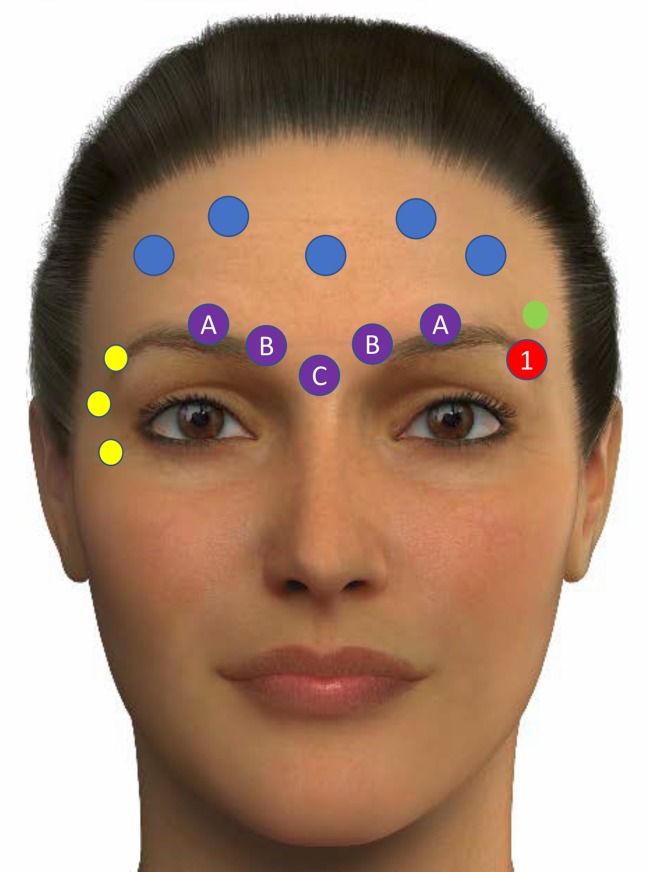
Different treatment points of the upper face. Adapted from de Maio et al. [26]. Yellow circles represent treatment with botulinum toxin A for crow feet lines. Blue circles represent treatment with botulinum toxin A for the forehead lines. Purple circles show the points for treatment of glabellar lines with botulinum toxin A. Red circle shows the point (E1) for lifting the tail of the eyebrow with low-density hyaluronic acid implant. Green circle shows the point for treating with high-density hyaluronic acid implant, the temporal region (T1). **a** Indicates one-third needle depth. **b** Indicates full-needle depth. **c** Indicates one-half needle depth. The images of this figure are an Allergan property, and consent was provided for their use in this publication

**Fig. 4:**
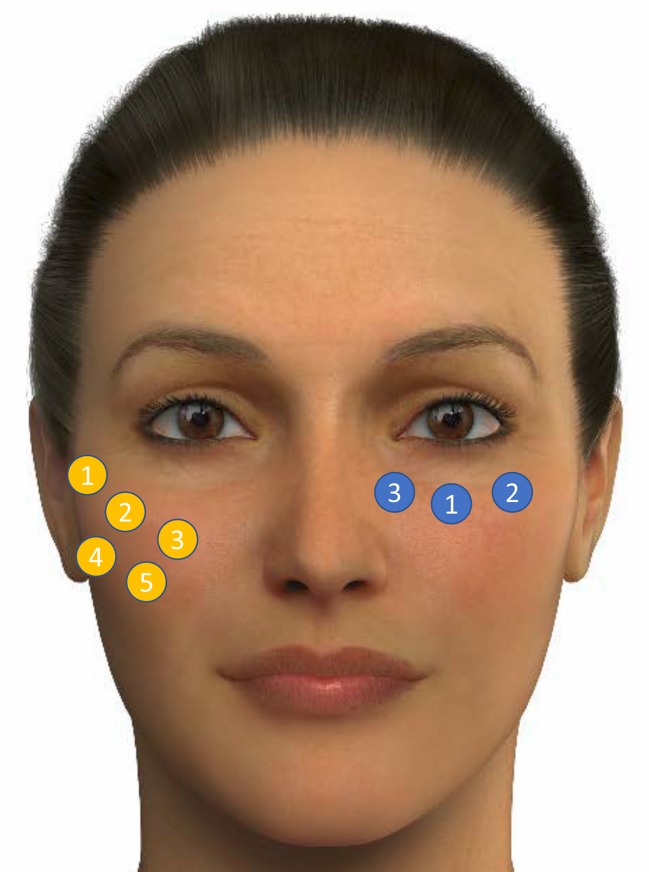
Different treatment points of the mid-face. Adapted from de Maio et al. [27]. Blue circles show the volume replacement along the lid–cheek junction: 1 = Tt 1; 2 = Tt2; 3 = Tt3. Low-density hyaluronic acid fillers implants with low density are injected at three main locations (two injections per location; up to six total aliquots). Orange circles represent the treatment points for volume replacement in the malar area by using deep supraperiosteum injections with a high-density hyaluronic acid filler at first three sites (1 = Ck1, 2 = Ck2, 3 = Ck3) per side and a small bolus and submalar area by using subcutaneous injections with a medium-density hyaluronic acid filler at the last two sites (4 = Ck4, 5 = Ck5). The images of this figure are an Allergan property, and consent was provided for their use in this publication

**Fig. 5:**
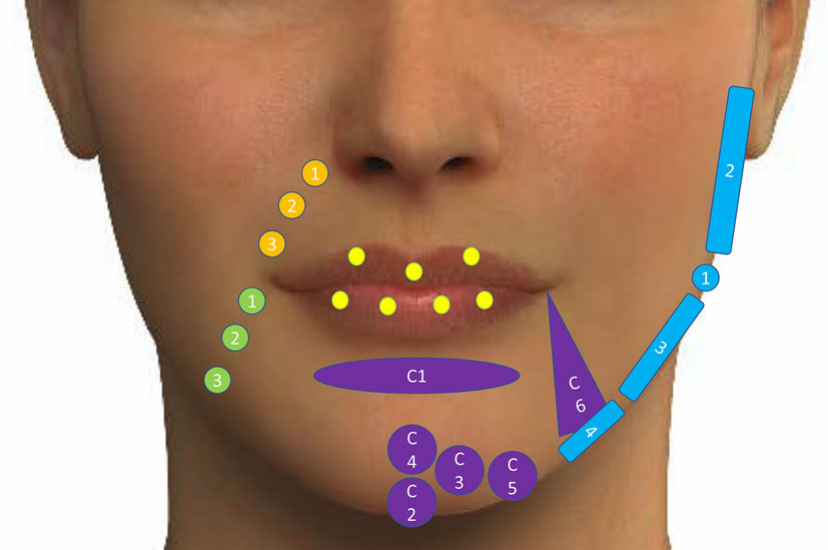
Different treatment points of the lower face. Adapted from de Maio et al. [28]. Yellow circles: treatment of lips with hyaluronic acid dermal fillers. Orange circles: treatment of the nasolabial fold (1 = Nl1, 2 = Nl2, 3 = Nl3). Purple figures: points to treat the chin with high-density hyaluronic acid filler. Green circles: points for the treatment of marionette lines (1 = M1, 2 = M2, 3 = M3). Blue figures: points to treat the jawline with high-density hyaluronic acid filler (1 = Jw1, 2 = Jw2, 3 = Jw3, 4 = Jw4). The images of this figure are an Allergan property, and consent was provided for their use in this publication

The different treatment points of the upper face, mid-face and lower face are shown in Figs. 3, 4 and 5, respectively.

## Results

These treatment strategies have been adapted to six different basic categories of facial shapes and are based on the author/authors clinical experience [30].

### Treatment Strategy According to the Facial Shape

Round face (Fig. 1b):

- Arched brows and tail eyebrows in upward mode with BoNTA in periorbital, frontal and glabellar regions.
- Cheek (Ck) and tail of the eyebrows (E) in upward mode with HA implant in Ck1, Ck2 and E1.
- HA implant in chin (C) with C1 and C2 (to achieve eversion of the chin).

**Fig. 6:**
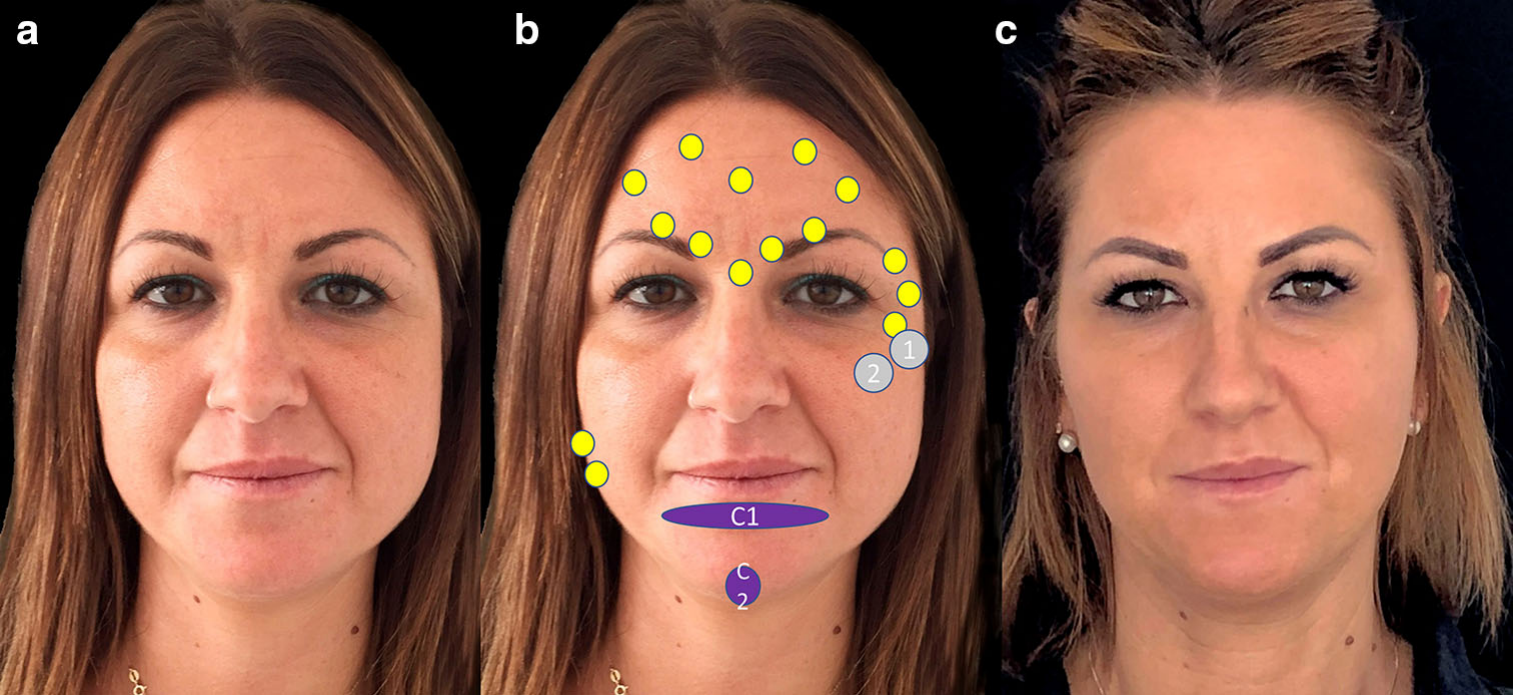
Patient with a round face before treatment (**a**), before treatment showing the treatment plan and after treatment (**c**). The treatment strategy was: hyaluronic acid: gray circles: 1 = CK1 (0, 4 ml) and 2 = Ck2 (0, 4 ml). Deep supraperiosteum injections with a high-density hyaluronic acid filler (VYC-20L) in upward mode, to ascending lateral cheekbones. Purple figures: C1 (1 ml) and C2 (0.6 ml) for getting chin eversion by using subcutaneous and deep injections with a high-density filler. Botulinum toxin A: yellow circles. Arched brows and tail eyebrows in upward mode in periorbital, frontal and glabellar regions 46 IU OnabotA in a superior third. Soften the angles of the jaw on the masseter muscles 50 IU in the lower third. The patient has provided a consent for using them in this publication

Figure 6 illustrates the technique and treatment effect of a patient with round face.

Square face (Fig. 1c):

- Light in the forehead and arched brows in upward mode with BoNTA in periorbital, frontal and glabellar regions with BoNTA.
- Cheek and tail of the eyebrows in upward mode with HA implant in Ck1, Ck2 and E1.
- Chin area with C1 and C2 (to achieve eversion of the chin).
- Soften the jaw (Jw) angles with BoNTA in masseter muscles.

**Fig. 7:**
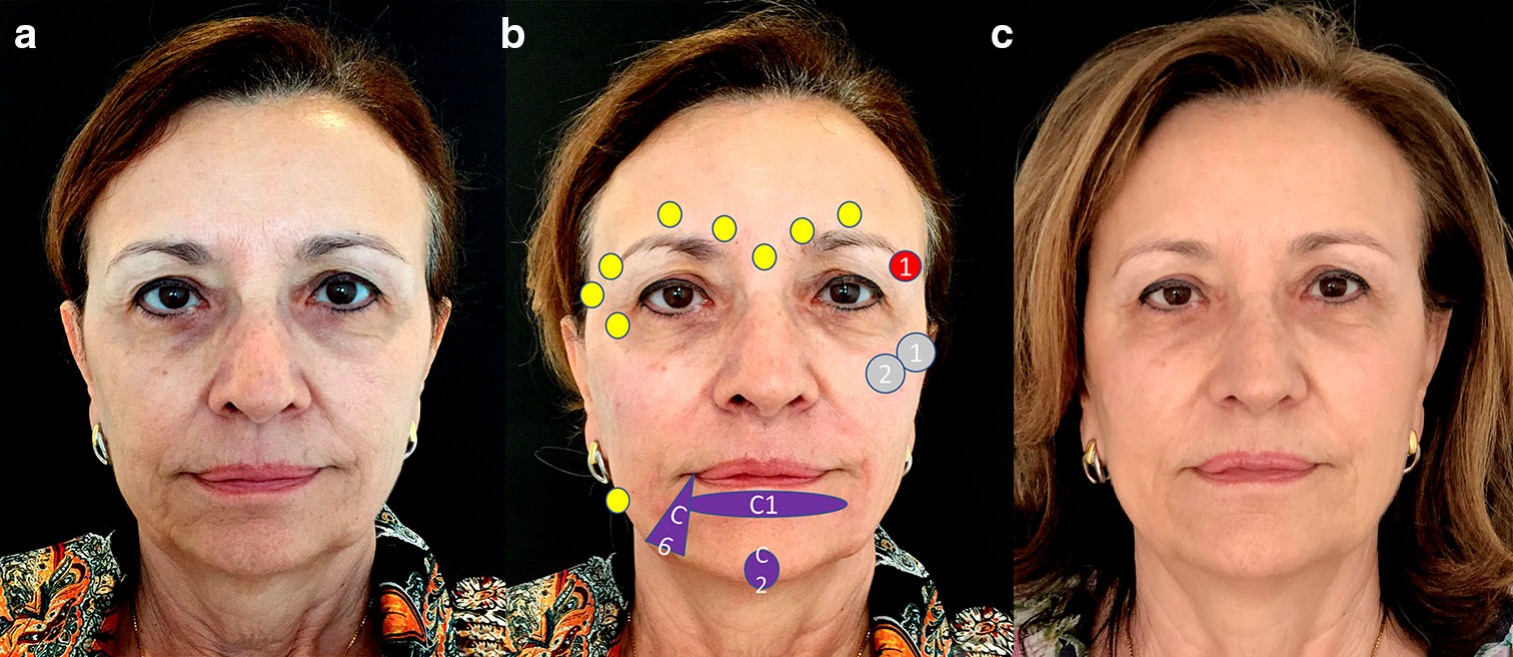
Patient with a square face before (**a**), immediately after treatment showing the treatment plan (**b**) and after the treatment (**c**). The treatment strategy was: hyaluronic acid: gray circles: 1 = CK1 (0, 6 ml) and 2 = Ck2 (0,4 ml). Deep supraperiosteum injections with a high-density hyaluronic acid filler (VYC-20L) in upward mode, to ascending lateral cheekbones. Purple figures: C1 (1 ml), C2 (0.6 ml) for getting chin eversion and C6 (1 ml) for the prejowl zone, using a subcutaneous and deep injections with a high-density filler. Red circle: 1 = E1 (0,2 ml) for lifting the tail of eyebrow, using a subcutaneous injection with a low-density filler. Botulinum toxin A: yellow circles. Arched brows and tail eyebrows in upward mode in periorbital and glabellar regions 36 IU in a superior third. Soften the angles of the jaw on the masseter muscles 40 IU OnabotA in the lower third. The patient has provided a consent for using them in this publication

Figure 7 shows the treatment administered and its effect in a patient with a square face.

Triangle face (Fig. 1d):

- HA implant in temporal (T) regions T1 and T2 and upward cheek areas with Ck1, Ck2 and Ck3. Lengthening of the chin areas C1 and C2.
- Light in glabellar, frontal and periorbital regions with BoNTA (to highlight the upper third and widen it).
- Soften the Jw angles with BoNTA in masseter muscles.

Inverted triangle face (Fig. 1e):

- Corrections with HA implant will try to give more importance to the middle and lower third of the face, treating cheek areas with Ck3, Ck4 and Ck5, as well as jaw (Jw) areas Jw1, Jw2 and Jw3.

**Fig. 8:**
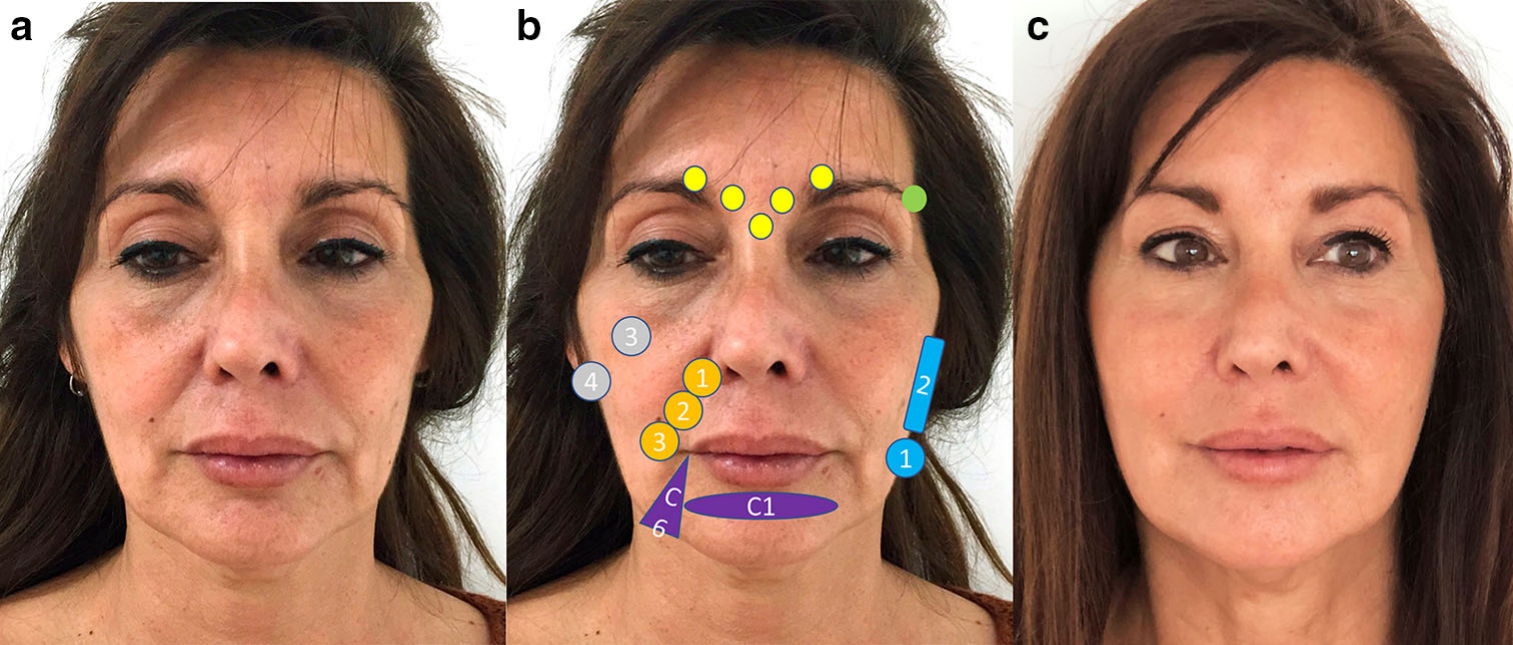
Patient with a combination between triangle and inverted triangle face before treatment (**a**), before treatment showing the treatment plan (**b**) and after the treatment (**c**). The treatment strategy was: botulinum toxin A: yellow circles, 20 IU OnabotA in the glabellar lines. Hyaluronic acid: green circle: T1 (1.4 ml) for the temporal fossa using deep supraperiosteum injections with a high-density hyaluronic acid filler (VYC-20L). Gray circles: 3 = Ck 3 (0.6 ml), 4 = Ck 4 (1 ml) for the malar and submalar area using a subcutaneous injection with a high-density hyaluronic acid filler. Blue figures: 1 = Jw 1 (0.4 ml), 2 = Jw 2 (1 ml) for the jaw line and mandibular angle using a subcutaneous injection with a medium-density hyaluronic acid filler. Orange circles: 1 = Nl1, 2 = Nl2, 3 = Nl3 (1 ml) for the nasolabial folds using subcutaneous injections with a medium-density hyaluronic acid filler. Purple figures: C1(1 ml) for the eversion of the chin and C6 (1 ml) for the prejowl zone using a subcutaneous and deep injections with a high-density hyaluronic acid filler. The patient has provided a consent for using them in this publication

Figure 8 presents the treatment strategy and its effect in a patient with a combination between triangle and inverted triangle face.

Rectangle face (Fig. 1f):

- Light in the glabellar and periorbital regions with BoNTA for highlighting the way of looking and widen this third of the face.
- Cheek areas Ck1 and Ck2 (horizontally) and chin areas C1 and C2 for sharpening the face.
- Soften the jaw angles with BoNTA in masseter muscles.

Oblong face (Fig. 1g):

- If empty temporal fossa, will inject in T1, in medial third Ck 1, Ck 2, Ck 3 (horizontally), Ck4, Ck 5 and highlight the jaw areas Jw 1, Jw 2 and Jw 3.
- Light in the glabellar, frontal and periorbital regions with BoNTA for highlighting the way of looking; flatten the eyebrow and widen the face.

**Fig. 9:**
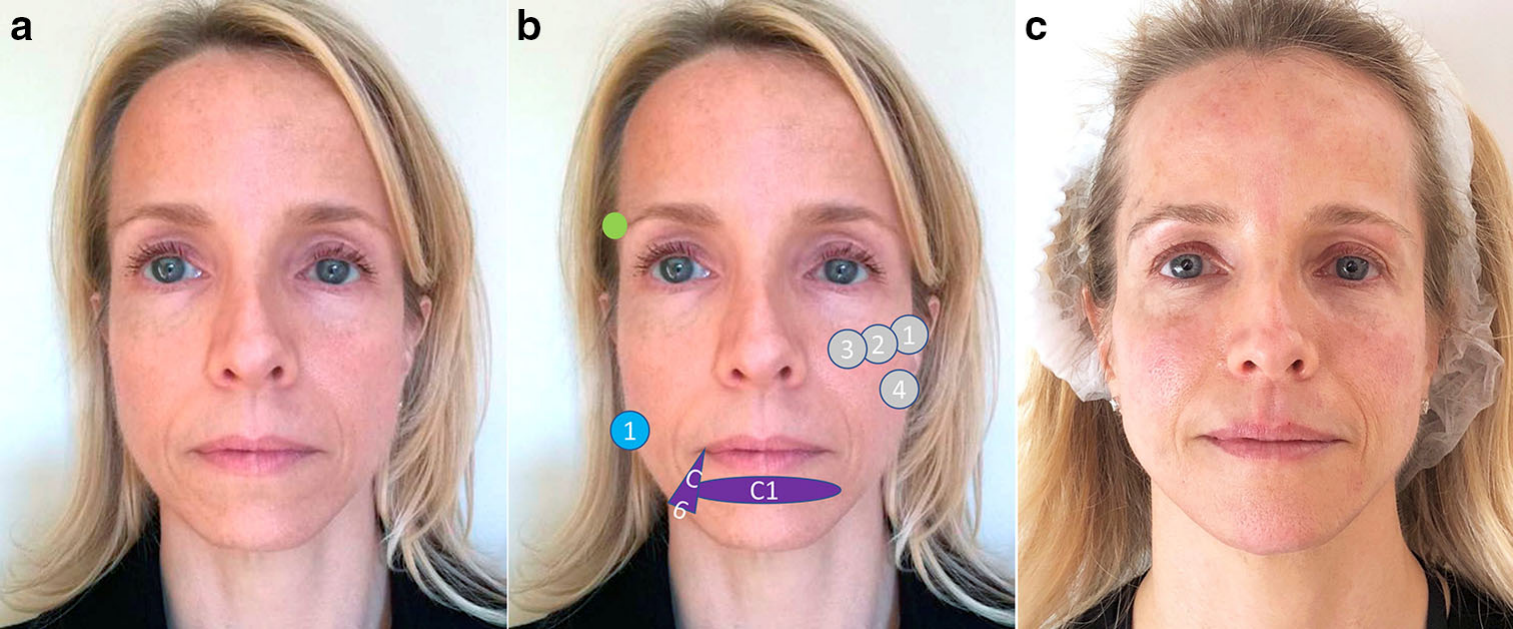
Patient with an oblong face before treatment (**a**), before treatment showing the treatment plan (**b**) and after the treatment (**c**). The treatment strategy was: hyaluronic acid: green circle: T1 (1.4 ml) for the temporal fossa using deep supraperiosteum injection with a high-density hyaluronic acid filler (VYC-20L). Gray circles: 1 = Ck 1, (0.2 ml), 2 = Ck 2 (0.4 ml), 3 = Ck 3 (0.6 ml) for the malar area in horizontal mode, using deep supraperiosteum injection with a high-density hyaluronic acid filler and Ck4 (1 ml) for the submalar area using subcutaneous injections with a medium-density hyaluronic acid filler. Blue circle: 1 = Jw 1 (0.2 ml) for the mandibular angle using deep supraperiosteum injection with a high-density hyaluronic acid filler. Purple figures: C1(1 ml) for the eversion of the chin and C6 (1 ml) for the prejowl area using a subcutaneous and deep injections with a high-density hyaluronic acid filler. The patient has provided a consent for using them in this publication

Figure 9 shows the treatment administered and its effect in a patient with an oblong face.

### Treatment Strategy According to the Axis

#### Vertical Axis

- We can elevate the outer canthus of the eye with Ck1 and raise the descended brows with E1.
- We can elongate the face with C1 and C2 to extend the lower third, if necessary.

#### Horizontal Axis

- It is possible to widen the lateral fifths, applying in T1.
- To stretch the distance of the medial fifths with glabellar BoNTA.
- Shorten the horizontal distance of the nasal wings to coincide with the inner canthus of the eye by injecting into the canine fossa Nl 1.
- Increase the lip (Lp) areas, with Lp1 and Lp3 to match the midline pupillary.

#### Anteroposterior Axis

Although severe skeletal malocclusion required, in many cases, a combined surgical approach [31] and, in mild cases, an aesthetic approach may be feasible.

##### Retrognathia

The dermal fillers should be used to provide the structure and support to the soft tissues.

- Lower third of the face or mandibular region: to enhance volume in the whole region with Lp1, Lp3 and Lp5 in the lower lips/M1, M2 and M3, in addition C1, C2, C3, C4, C5 (in male patient) and C6 and finishing treating Jw1 and Jw 3.

##### Prognathism

- Mid-third of the face or maxilla region: to enhance volume in the whole region with Ck 3, Nl1, Nl2 and Nl3 plus Lp1, Lp2, Lp3, Lp4 and Lp7.

### Asymmetries

- Mid-third:
  - Filling with a mid-density hyaluronic acid filler in Ck1 and Ck2 (a few millimeters above that in the contralateral side) and Nl1 in the short and full side.
  - High-density hyaluronic acid filler, injected deeply, in Ck1, Ck2, Ck3 and Ck4 in the contralateral hemiface (narrower and longer side).
- Lower third: a high-density hyaluronic acid filler, injected deeply, in Jw1 and Jw2 on the thin and long side and Jw3, Jw4, C1 and C2 in the short and full side.

Regarding the safety profile of our strategies, the incidence of complications was low and, in all the cases, was mild (edema, erythema, and local ecchymosis), of limited duration, and was resolved without sequela.

## Discussion

The number of patients asking for aesthetic procedures has been exponentially increased over the last several years [10–12]. In most cases, these aesthetic procedures are elective and, therefore, the patient’s satisfaction is first and foremost. However, once the patient’s treatment goals have been discussed and agreed, the specialist has the responsibility to discuss in depth which treatment approach is best suited to the patient’s needs, as well as the limitations of such approach in general.

Because beauty is not an exact science and critically depends on social, cultural and ethnic factors [1, 2, 5, 6], the aesthetic treatment approach should not be focused on producing “common perfect faces,” but rather to enhance those features that define the patient’s face. To offer the best possible outcomes, the aesthetic specialist should think about comprehensive approaches looking at the whole face and at different tissue depths to determine the appropriate treatment approach [32].

Our strategies provide a whole treatment approach based on a specific diagnostic methodology that pays special attention to facial morphology. As was stated in Methods, these strategies are based on The MD codes^®^, which favors the use of a methodical technique in all treatment settings and for all facial areas [26–28].

The goal of our treatment strategies is to enhance those features of the face personality that may give it an individualized touch of beauty or distinction, as well as to soften or cover up those aspects that can be considered as unattractive. However, it is essential to be careful to avoid creating “standard faces” that might remove important aspects of the patient personality.

Although with these approaches we also try to minimize the impact of slight asymmetries on the final outcome, they have not been designed for correcting moderate to severe asymmetries. The current literature has highlighted different causal factors as responsible for the development of facial asymmetries, including congenital, pathological, traumatic, functional or developmental causal factors [25, 33, 34]. Haraguchi et al. [25] reported that in minor facial asymmetry, the right hemiface is wider than the left hemiface with a chin deviation to the left side.

Additionally, Severt and Proffit reported frequencies of facial laterality of 5, 36 and 74% in the upper, middle and lower thirds of the face [24].

Paying special attention to facial asymmetry provides an important reference point in the patient face, because it allows to compare the wide/full and short facial side versus the narrow one and long [35].

Because less invasive aesthetic procedures for facial beautification and enhancement are evolving continuously, it is necessary to standardize their management providing strategies that prevent or reduce the incidence of complications [13, 15, 16, 35].

Although hyaluronic acid implants and BoNTA treatments are generally regarded as safe, unanticipated events and adverse outcomes can occur with these agents [13, 14, 16]. A careful patient selection, an adequate product and technique, correct aseptic approach, an exhaustive knowledge of facial anatomy coupled with constant awareness of the early signs of vascular compromise are required for proper patient management [13, 15, 16, 35].

The incidence and type of complications found with these approaches did not differ from those previously reported [13, 14, 16]. In all the cases, the adverse events were mild and were successfully resolved.

## Conclusions

The current article presented the personal experience of the authors on a specific subject, and this fact should be considered when interpreting data from this paper. This paper is only informative and addressed to the specialists.

For obtaining optimal outcomes, it is necessary for specialists to have a thorough understanding of facial anatomy as well as an appreciation of the aesthetic idea. As other esthetic treatments, facial contouring should be focused on patient needs and to select a specific aesthetic approach according to different facial shapes.

Although recent advances, including more versatile facial fillers, refined implantation techniques and adoption of a global facial approach, have contributed to improved patient outcomes and increased patient satisfaction, the continuous evolving of the esthetic techniques makes it necessary to implement good training programs.

Last but not least, it is essential to have a good understanding of the potential associated complications, because it will help the specialist to take the necessary precautions to prevent them, and if they ever arise, to be able to deal with them effectively.

There is a need for high-quality prospective studies that evaluate the efficacy and safety of the different treatment strategies.
